# RIRS with FV-UAS vs. ESWL for the management of 1–2 cm lower pole renal calculi in obese patients: a prospective study

**DOI:** 10.3389/fmed.2024.1464491

**Published:** 2024-10-30

**Authors:** Chao Men, Miao Xu, Si-cong Zhang, Qing Wang, Jie Wu, Yun-Peng Li

**Affiliations:** ^1^Department of Urology, Chifeng Cancer Hospital, Chifeng, China; ^2^Department of Urology, The Affiliated Jiangning Hospital of Nanjing Medical University, Nanjing, China; ^3^Department of Urology, The Second Affiliated Hospital of Nanjing Medical University, Nanjing, China; ^4^Department of Clinical Laboratory, Nanjing First Hospital of Nanjing Medical University, Nanjing, China

**Keywords:** lower pole renal calculi, obesity, flexible ureteroscopy, extracorporeal shock wave lithotripsy, combination therapy

## Abstract

**Objective:**

To evaluate the efficacy and safety of retrograde intrarenal surgery (RIRS) combined with flexible vacuum-assisted ureteral access sheath (FV-UAS) versus extracorporeal shock wave lithotripsy (ESWL) for the management of 1–2 cm lower pole renal calculi (LPC) in obese patients.

**Patients and methods:**

This prospective, randomized study included 149 obese patients with 1–2 cm LPC. Patients were allocated into two groups: 76 patients underwent RIRS with FV-UAS, and 73 patients received ESWL. The parameters assessed included stone-free rate (SFR), retreatment rate, complications, operative time, and pain intensity measured by the visual analog scale (VAS). Stone-free status was defined as the absence of stones on computed tomography or residual fragments smaller than 4 mm at 4 weeks post-procedure.

**Results:**

The baseline characteristics of the two groups were comparable. The SFR was significantly higher in the RIRS group, reaching 86.8%, compared to 63.0% in the ESWL group (*p* = 0.034). Furthermore, the retreatment rate was significantly lower in the RIRS group, at 5.2%, versus 24.7% in the ESWL group (*p* < 0.001). The average operative time for RIRS was notably longer, at 65.3 ± 6.4 min, compared to 25.3 ± 7.8 min for ESWL (*p* < 0.001). The complication rates were 9.2% for the RIRS group and 6.8% for the ESWL group, with no statistically significant difference (*p* = 0.326). All complications were classified as Grade I or II according to the modified Clavien classification system. No significant differences were observed between the two groups regarding pain VAS scores and the composition of the stones.

**Conclusion:**

RIRS with FV-UAS demonstrated superior efficacy, evidenced by a higher SFR and reduced retreatment rates compared to ESWL, despite a longer operative duration. Both treatment modalities showed comparable safety profiles. RIRS with FV-UAS emerges as a viable, effective, and reproducible intervention for managing 1–2 cm LPC in obese patients, providing significant clinical advantages.

## Introduction

The World Health Organization defines obesity as having a body mass index (BMI) of 30 kg/m^2^ or higher (1). This condition represents a major global health issue, given its increasing prevalence and the numerous comorbidities linked to it, such as kidney stone disease (KSD). The management of kidney stones in obese patients presents unique challenges due to anatomical and physiological differences (2), which can impact the efficacy and safety of various treatment modalities.

Lower pole renal calculi (LPC), a common type of kidney stone, are particularly challenging to treat due to their location within the kidney (3). ESWL and RIRS are frequently utilized minimally invasive methods for managing renal calculi (4). ESWL uses shock waves to break stones into smaller fragments (5), allowing them to be passed naturally. However, its effectiveness can be limited in obese patients due to the increased distance between the skin and the stone, and the lower effectiveness in treating LPC (6). On the other hand, RIRS (7) involves the use of a flexible ureteroscope to directly visualize and fragment the stones, which can be more effective but also more technically demanding and time-consuming.

Recent technological advancements, such as the flexible vacuum-assisted ureteral access sheath (FV-UAS), aim to enhance the outcomes of RIRS by aiding in the efficient removal of stone fragments and mitigating intrarenal pressure (8). Despite these advancements, there is a paucity of comparative data on the efficacy and safety of RIRS with FV-UAS versus ESWL specifically in obese patients with LPC. In spite of these technological progressions, comprehensive comparative studies evaluating the effectiveness and safety of RIRS with FV-UAS versus ESWL in obese patients with LPC remain markedly insufficient.

This study aims to evaluate and compare the efficacy and safety of RIRS combined with FV-UAS and ESWL for the management of 1–2 cm LPC in obese patients. By assessing key outcomes such as stone-free rate (SFR), retreatment rate, complications, operative time, and pain VAS score, this study seeks to provide insights into the most effective treatment approach for this patient population.

## Materials and methods

### Inclusion and exclusion criteria

Inclusion criteria: (1) Adult patients aged between 18 and 65 years. (2) Presence of lower pole renal calculi with a size ranging from 1 to 2 cm. (3) Body mass index (BMI) exceeding 30 kg/m^2^. (4) Normal renal function and anatomy of the renal tract.

Exclusion criteria: (1) Uncontrollable urinary tract infections (UTI). (2) Severe cardiovascular diseases, end-stage renal failure, or severe coagulation disorders. (3) Pregnancy or presence of bilateral kidney stones. (4) Prior history of ipsilateral extracorporeal shock wave lithotripsy (ESWL) or retrograde intrarenal surgery (RIRS).

### Randomization

Following the acquisition of informed consent from the participants, research personnel at our institution employed a computer-generated random number coding table to facilitate the allocation of eligible patients. The random assignments were securely contained within sealed envelopes. Participants were randomized in a 1:1 ratio to ensure methodological rigor.

### Participants and preoperative preparation

**Figure 1:**
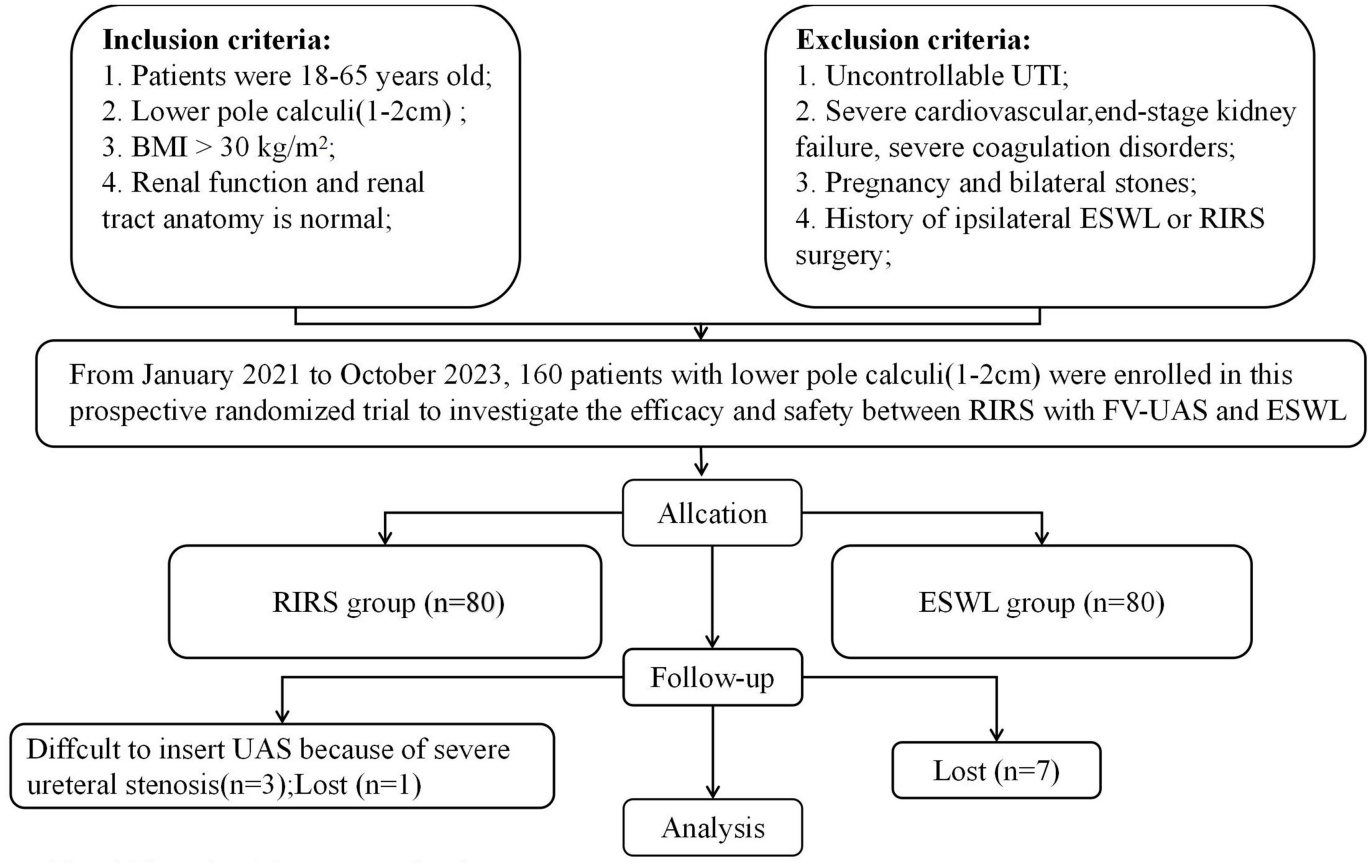
Flowchart for case selection.

The study included 149 patients, 76 in the RIRS group and 73 in the ESWL group, based on power analysis performed to estimate the sample size (Figure 1). Prior to the initiation of treatment, all participants underwent a thorough pre-treatment evaluation, which included the collection of demographic data, a detailed medical history, physical examination, routine laboratory investigations, and radiological assessments. Non-contrast computed tomography (CT) scans were employed to ascertain the characteristics of the renal calculi, with the stone size being defined by the maximum diameter of the calculi.

### ESWL and RIRS techniques

An electromagnetic lithotripter (Dornier Lithotriptor S, Dornier MedTech GmbH, Germering, Germany) was utilized for all extracorporeal shock wave lithotripsy (ESWL) procedures, chosen for its proven effectiveness in renal calculi fragmentation. Energy levels for the shock waves were precisely controlled within a range of 12–14 kV. Each ESWL session involved the administration of up to 3,000 shock waves, delivered at a frequency of 60–90 shocks per minute, until complete fragmentation of the renal calculi was achieved. Following each ESWL session, patients were scheduled for a follow-up evaluation 1 week later. This assessment included kidney, ureter, and bladder (KUB) radiographs as well as renal ultrasonography to evaluate the extent of stone fragmentation and detect any potential renal obstruction. In cases where stone fragmentation was deemed insufficient, additional ESWL sessions were performed. A maximum of three ESWL sessions were permitted per patient. If three sessions failed to achieve adequate stone breakage, patients were transitioned to alternative treatment modalities.

**Figure 2:**
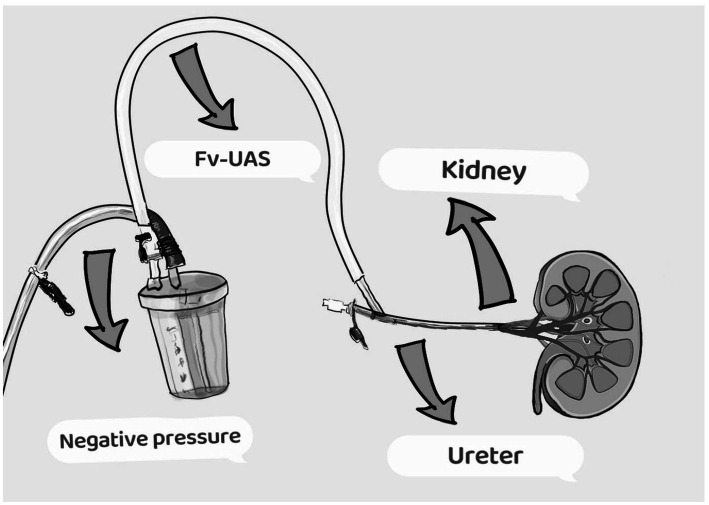
The combination of RIRS and FV-UAS.

The procedure for the FV-UAS group commenced with the administration of general anesthesia and positioning of patients in the lithotomy position to facilitate retrograde endoscopic access. Initial access to the ureter was achieved via the introduction of a Zebra guidewire using a rigid ureteroscope. Over this guidewire, an 11/13 Fr FV-UAS (36 cm for females, 46 cm for males) was advanced. A flexible ureteroscope was then utilized to identify renal calculi. The FV-UAS was then directed to the target calculus under fURS visualization, and stone fragmentation was performed using a 200 μm holmium laser fiber set at 0.4–0.6 J and a frequency of 30–40 Hz. Central negative pressure suction (85–90 cm H_2_O) ensured balanced fluid dynamics and clear visibility. Continuous suction of stone fragments was facilitated through the repeated insertion and withdrawal of the fURS, maintaining an irrigation flow rate of 80 mL/min. Upon concluding the fragmentation, the collecting system was reassessed for residual stone fragments, and both the FV-UAS and fURS were carefully withdrawn while monitoring for ureteral injuries (Figure 2). A 6 Fr double-J stent was placed in each patient to maintain ureteral patency. In instances of severe ureteral stenosis or distortion, balloon dilation was attempted; if unsuccessful, a double-J stent was implanted to promote gradual ureteral dilation.

### Postoperative evaluation

The primary outcome measure was the stone-free rate (SFR) at 4th week, defined as the absence of detectable stones on imaging studies post-treatment. Secondary outcomes included the retreatment rate, incidence of complications, operative time, and pain assessment using the visual analog scale (VAS).

### Statistical analysis

SPSS v.22.0 for Windows (IBM Corp., Armonk, NY, United States) was used for statistical analysis. Demographic characteristics, duration of follow-up, and clinical outcomes were compared between the two groups using the independent samples *t*-test; other clinical characteristics were compared between the two groups using the chi-square test. *p* < 0.05 was considered a significant difference.

## Results

The study enrolled 160 eligible patients who were randomized into two groups: 80 patients in the RIRS group and 80 patients in the ESWL group. During the treatment and follow-up phase, 11 patients withdrew, resulting in 149 patients being analyzed for the primary outcome (Figure 1).

The demographic and clinical characteristics of the two groups were comparable, with no statistically significant differences observed (Table 1). Specifically, there were no notable differences in age, gender, and BMI. Additionally, the groups were matched in terms of medical history, including hypertension and diabetes, as well as stone burden, Hounsfield units, skin-to-stone distance, lower pole anatomy, stone location, and preoperative urine culture results (all *p* > 0.05).

**Table 1:** Demographic and clinical characteristics.

Variables, mean ± SD or *n* (%)	RIRS group (*n* = 76)	ESWL group (*n* = 73)	*p*
BMI (kg/m^2^)	32.7 ± 1.5	32.8 ± 1.4	0.640
Age (year)	49.2 ± 8.6	48.7 ± 9.0	0.730
**Gender**			
Male	30 (39.5%)	28 (38.4%)	—
Female	46 (60.5%)	45 (61.6%)	0.890
Hypertension history	10 (13.2%)	12 (16.4%)	0.475
Diabetes history	9 (14.5%)	10 (13.7%)	0.520
Mean stone size (mm)	15.4 ± 3.2	16.1 ± 3.4	0.275
Stone burden (mm^2^)	231.5 ± 49.0	228.3 ± 48.7	0.693
Hounsfield units	980.45 ± 150.32	960.38 ± 145.27	0.310
Skin-to-stone distance (mm)	110.5 ± 14.9	110.9 ± 13.7	0.725
**Lower-pole anatomy**			
Infundibular-pelvic angle	49.5 ± 2.8	50.2 ± 3.1	0.320
Infundibular length (mm)	22.3 ± 2.1	23.1 ± 1.8	0.325
Infundibular width (mm)	5.8 ± 1.5	5.6 ± 1.3	0.680
**Stone position**			
Left side	31 (40.7%)	29 (39.7%)	-
Right side	45 (59.3%)	44 (60.3%)	0.290
Urine culture (positive)	6 (11.3%)	5 (8.6%)	0.365

BMI, body mass index; SD, standard deviation. ^*^p < 0.05 and ^**^p < 0.01.

Table 2 summarizes the treatment and post-treatment data. The stone-free rate in the RIRS group was significantly higher compared to the ESWL group [86.8% vs. 63.0% (*p* = 0.034)]. The ESWL group exhibited a significantly higher retreatment rate due to the necessity for multiple sessions in 24.7% of patients (*p* < 0.001). Additionally, the mean operation time was notably longer in the RIRS group compared to the ESWL group (65.3 ± 6.4 vs. 25.3 ± 7.8 min, *p* < 0.001). The mean pain scores between the two groups were similar (4.1 ± 0.5 vs. 3.8 ± 0.4, *p* = 0.054). Postoperative complications were assessed using the modified Clavien classification system, revealing no significant difference in overall complication rates between the RIRS and ESWL groups (*p* > 0.05). In the RIRS group, two cases of postoperative fever were reported, whereas the ESWL group experienced four cases. All patients with postoperative fever showed improvement following the administration of antipyretic medications. Additionally, two patients in the RIRS group experienced urine leakage; however, no such incidents were reported in the ESWL group. The urine leakage in the RIRS group resolved spontaneously without the necessity for medical intervention. Furthermore, one patient in the RIRS group required a blood transfusion, and two patients received total parenteral nutrition. In the ESWL group, one patient also necessitated a blood transfusion. Similarly, the analysis of stone composition revealed no significant differences between the two groups (*p* > 0.05).

**Table 2:** Clinical outcomes and complications.

Variables, mean ± SD or *n* (%)	RIRS group (*n* = 76)	ESWL group (*n* = 73)	*p*
Operative time (min)	65.3 ± 6.4	25.3 ± 7.8	*p* < 0.001
Pain VAS score (range 1–10)	4.1 ± 0.5	3.8 ± 0.4	0.054
Total SFR at 4th week	66 (86.8)	46 (63.0)	0.034
Retreatment rate	4 (5.2)	18 (24.7)	*p* < 0.001
Complications	7 (9.2)	5 (6.8)	0.326
**Modified Clavien classification**			
Grade I	4 (5.3)	4 (5.4)	—
Grade II	3 (3.9)	1 (1.3)	—
**Stone analysis**			
Uric acid	20 (26.3)	21 (28.8)	0.703
Calcium oxalate	42 (55.3)	39 (53.4)	0.845
Carboapatite	14 (18.4)	13 (17.8)	0.981

*p < 0.05 is considered statistically significant. SD, standard deviation. SFR, stone-free rates.*

## Discussion

Numerous cohort studies have substantiated that individuals classified as obese exhibit an elevated risk of developing kidney stone disease (KSD) (9, 10). The National Health and Nutrition Examination Survey (NHANES), a cross-sectional study, corroborated a higher prevalence of KSD among obese individuals compared to those of normal weight (11, 12). According to the European Association of Urology (EAU) guidelines, shock wave lithotripsy (SWL), percutaneous nephrolithotomy (PCNL), and flexible ureteroscopy (fURS) are established standard therapeutic approaches for the management of renal calculi (13). SWL operates by employing high-energy acoustic waves to externally fragment stone, thereby facilitating their expulsion through the urinary tract. Despite demonstrating lower stone-free rates (SFR) and necessitating higher retreatment rates, SWL remains a favored intervention due to its noninvasive nature and high acceptance among healthcare professionals and patients alike (14).

However, the success rate of SWL is significantly reduced in obese patients (15). This attention in success rate is primarily attributed to factors such as increased skin to stone distance, which enhances shock wave absorption, composites in stone localization, and the radiolucency of urate stones, all of which affect the effective targeting of the shock wave beam. Additionally, an elevated BMI is associated with a heightened probability of residual stone fragments post SWL, compared to achieving a stone free status (16). This association was further corroborated by Mezentsev (17), who documented a stone free rate of 73% in obese patients. A BMI exceeding 30 kg/m^2^ is also correlated with an elevated risk of renal hematoma (18), potentially attributable to the inappropriate utilization of high-energy shock waves.

Currently, a variety of minimally invasive techniques have demonstrated efficacy in managing LPC measuring between 10 to 20 mm. The feasibility of RIRS has been significantly augmented by ongoing advancements in flexible ureteroscopes (fURS), laser technology, and ureteral access sheaths. FURS is a minimally invasive endoscopic procedure that employs lasers to fragment kidney stones located within the urinary tract (19), theoretically circumventing the influence of obesity. Contemporary complication rates associated with fURS are approximately 9%, with severe complications (Clavien Grade >III) comprising less than 1% (20).

Obese patients present unique challenges in the management of kidney stones, particularly during surgical interventions, due to a higher risk of perioperative complications such as bleeding, infection, and respiratory issues, alongside increased abdominal pressure which complicates the surgical field and often results in longer operative times. Anesthesia administration is similarly complicated by factors like potential respiratory compromise and a heightened risk of obstructive sleep apnea, leading to longer recovery times and increased monitoring needs. Managing intrarenal pressure (IRP) (21) during procedures like retrograde intrarenal surgery (RIRS) is particularly difficult, as elevated intra-abdominal pressure affects fluid dynamics during irrigation and drainage, complicating visualization and increasing complication risks. Notably, factors such as stone size, location, obesity, and the infundibulopelvic angle significantly influence the stone-free rate (SFR) (22). To address these challenges, advancements like the flexible access sheath (FV-UAS) (23) have been developed, which passively bends with the flexible ureteroscope (fURS) to navigate the ureteropelvic junction and access the renal pelvis and calices more effectively. This device facilitates free drainage of irrigation fluid, improving visualization and preventing harmful elevations in IRP. Based on the *in vitro* findings of Chen et al. (24), we have calibrated fluid irrigation to 80 mL/min and maintained negative pressure at 85–90 cmH_2_O, keeping IRP below 10 cmH_2_O and allowing the renal pelvis to remain semi-saturated, thereby enhancing procedural efficiency. Consequently, we have adopted a novel technique that integrates fURS with FV-UAS to improve treatment outcomes for large calculi (LPC) measuring 10–20 mm, effectively mitigating risks traditionally associated with percutaneous renal surgery while achieving a high SFR, thereby enhancing both safety and efficacy in managing kidney stones in obese patients.

In our study, the primary outcomes demonstrated a significantly higher SFR and a lower retreatment rate following RIRS, without a corresponding increase in complications when compared to the ESWL. Several factors elucidate these findings. Firstly, RIRS presents distinct advantages over ESWL, particularly due to the capability of laser energy in RIRS to fragment stones irrespective of their chemical composition or density. Furthermore, the FV-UAS in RIRS ensures direct entry into the target calculi. The suction generated by negative pressure through the FV-UAS enhances the removal of stone fragments, thereby improving the efficiency of the procedure. Conversely, the efficacy of ESWL is contingent upon several variables including patient obesity, stone density, chemical composition, and the skin-to-stone distance (25, 26). Notably, the SFR in RIRS is predominantly influenced by stone size, as indicated by Grasso and Ficazzola (27), who reported SFR of 82, 71, and 65% for stone sizes <1 cm, 1–2 cm, and >2 cm, respectively.

However, the incidence of complications was higher after RIRS than after ESWL, reflecting the more invasive nature of RIRS. Nonetheless, this difference was not statistically significant, and the severity of complications, as classified by the modified Clavien system, remained comparable between both groups. All complications were manageable with medical treatment. There was no significant difference in pain perception among patients, although RIRS required a significantly longer surgical time compared to ESWL. These findings highlight the superior efficacy and effectiveness of RIRS in achieving higher stone-free rates and reducing the necessity for retreatment when compared to ESWL. This is achieved despite the longer procedural duration and similar complication profiles.

This study does have some limitations that warrant acknowledgment. Firstly, the patient cohort consists solely of individuals with primary or secondary obesity, with no inclusion of cases of morbid obesity (BMI >40 kg/m^2^). This constrains the applicability of our results to the wider population, particularly to individuals with more severe obesity. Secondly, the follow-up period was relatively short, which could potentially influence the long-term assessment of outcomes such as recurrence rates and long-term complications. A longer follow-up period would provide a more comprehensive understanding of the durability of the stone-free state and the overall effectiveness of the procedures. Thirdly, this was a single-center study characterized by a small sample size. The restricted number of participants may diminish the statistical power of the research and hinder the detection of subtle differences between the groups. Additionally, single-center data may not be representative of broader clinical practices and outcomes in different settings.

## Conclusion

The study provides valuable insights into the comparative efficacy and safety of RIRS and ESWL, highlighting the superior stone-free rates and reduced retreatment rates associated with RIRS, alongside a comparable complication profile. Nevertheless, further research involving larger, multi-center cohorts with extended follow-up periods is necessary to validate these findings and to better understand the long-term benefits and risks associated with these treatment methods.

## Data Availability

The original contributions presented in the study are included in the article/supplementary material, further inquiries can be directed to the corresponding author.
